# Mutation Spectrum in TPO Gene of Bangladeshi Patients with Thyroid Dyshormonogenesis and Analysis of the Effects of Different Mutations on the Structural Features and Functions of TPO Protein through* In Silico* Approach

**DOI:** 10.1155/2019/9218903

**Published:** 2019-02-24

**Authors:** Mst. Noorjahan Begum, Md Tarikul Islam, Shekh Rezwan Hossain, Golam Sarower Bhuyan, Mohammad A. Halim, Imrul Shahriar, Suprovath Kumar Sarker, Shahinur Haque, Tasnia Kawsar Konika, Md. Sazzadul Islam, Asifuzzaman Rahat, Syeda Kashfi Qadri, Rosy Sultana, Suraiya Begum, Sadia Sultana, Narayan Saha, Mizanul Hasan, M. A. Hasanat, Hurjahan Banu, Hossain Uddin Shekhar, Emran Kabir Chowdhury, Abu A. Sajib, Abul B. M. M. K. Islam, Syed Saleheen Qadri, Firdausi Qadri, Sharif Akhteruzzaman, Kaiissar Mannoor

**Affiliations:** ^1^Laboratory of Genetics and Genomics, Institute for Developing Science and Health Initiatives (ideSHi), Mohakhali, Dhaka 1212, Bangladesh; ^2^Department of Genetic Engineering and Biotechnology, University of Dhaka, Dhaka 1000, Bangladesh; ^3^Department of Biochemistry and Molecular Biology, University of Dhaka, Dhaka 1000, Bangladesh; ^4^Infectious Diseases Laboratory, Institute for Developing Science and Health Initiatives (ideSHi), Mohakhali, Dhaka 1212, Bangladesh; ^5^The Red-Green Research Centre (RGRC), 218 Elephant Road, Dhaka 1205, Bangladesh; ^6^Nuclear Medicine and Allied Sciences, Bangabandhu Sheikh Mujib Medical University (BSMMU), Shahbag, Dhaka 1000, Bangladesh; ^7^Department of Biochemistry and Molecular Biology, Jagannath University, Dhaka, Bangladesh; ^8^Department of Paediatric Medicine, KK Women's and Children's Hospital, 100 Bukit Timah Road, Singapore; ^9^Bangladesh University of Health Sciences, Bangladesh; ^10^Department of Peadiatrics Endocrinology, Bangabandhu Sheikh Mujib Medical University (BSMMU), Shahbag, Dhaka 1000, Bangladesh; ^11^Pediatric Neurology, National Institute of Neurosciences & Hospital, Dhaka 1207, Bangladesh; ^12^Department of Endocrinology, Bangabandhu Sheikh Mujib Medical University (BSMMU), Shahbag, Dhaka 1000, Bangladesh; ^13^Department of Enteric and Respiratory Infectious Diseases, Infectious Diseases Division, International Centre for Diarrhoeal Disease Research, Mohakhali, Dhaka, Bangladesh

## Abstract

Although thyroid dyshormonogenesis (TDH) accounts for 10-20% of congenital hypothyroidism (CH), the molecular etiology of TDH is unknown in Bangladesh. Thyroid peroxidase (TPO) is most frequently associated with TDH and the present study investigated the spectrum of TPO mutations in Bangladeshi patients and analyzed the effects of mutations on TPO protein structure through* in silico* approach. Sequencing-based analysis of TPO gene revealed four mutations in 36 diagnosed patients with TDH including three nonsynonymous mutations, namely, p.Ala373Ser, p.Ser398Thr, and p.Thr725Pro, and one synonymous mutation p.Pro715Pro. Homology modelling-based analysis of predicted structures of MPO-like domain (TPO_142-738_) and the full-length TPO protein (TPO_1-933_) revealed differences between mutant and wild type structures. Molecular docking studies were performed between predicted structures and heme. TPO_1-933_ predicted structure showed more reliable results in terms of interactions with the heme prosthetic group as the binding energies were -11.5 kcal/mol, -3.2 kcal/mol, -11.5 kcal/mol, and -7.9 kcal/mol for WT, p.Ala373Ser, p.Ser398Thr, and p.Thr725Pro, respectively, implying that p.Ala373Ser and p.Thr725Pro mutations were more damaging than p.Ser398Thr. However, for the TPO_142-738_ predicted structures, the binding energies were -11.9 kcal/mol, -10.8 kcal/mol, -2.5 kcal/mol, and -5.3 kcal/mol for the wild type protein, mutant proteins with p.Ala373Ser, p.Ser398Thr, and p.Thr725Pro substitutions, respectively. However, when the interactions between the crucial residues including residues His239, Arg396, Glu399, and His494 of TPO protein and heme were taken into consideration using both TPO_1-933_ and TPO_142-738_ predicted structures, it appeared that p.Ala373Ser and p.Thr725Pro could affect the interactions more severely than the p.Ser398Thr. Validation of the molecular docking results was performed by computer simulation in terms of quantum mechanics/molecular mechanics (QM/MM) and molecular dynamics (MD) simulation. In conclusion, the substitutions mutations, namely, p.Ala373Ser, p.Ser398Thr, and p.Thr725Pro, had been involved in Bangladeshi patients with TDH and molecular docking-based study revealed that these mutations had damaging effect on the TPO protein activity.

## 1. Introduction

Congenital hypothyroidism (CH) is defined as insufficient production of thyroid hormone at birth [1]. The global frequency of CH is 1 in 3000-4000 whereas a pilot study in Bangladesh reported an incidence rate of 1 in 1300 [2, 3]. Thyroid dyshormonogenesis (TDH) which results due to defect in the pathway of thyroid hormone biosynthesis accounts for 10 to 20% cases of CH [4]. Till now, mutations in seven genes, namely, NIS (sodium iodine symporter, SLC5A5), PDS (Pendrin or SLC26A4), TPO (thyroid peroxidase), TG (thyroglobulin), IYD (iodotyrosine deiodinase, DEHAL1), DUOX2, and DUOXA2 (dual oxidase), have been reported to be involved in pathogenesis of TDH [5]. However, mutations in TPO gene which may result in total iodide organification defect (TIOD) or partial iodide organification defect (PIOD) have been described as the most common forms of TDH, and to date more than 60 different mutations have been described [6, 7].

TPO is the major enzyme in thyroid hormone biosynthesis and it catalyzes both iodination and coupling of iodotyrosine residues in TG (thyroglobulin). It is a glycosylated hemeprotein of 110 kDa bound at the apical membrane of thyrocytes [8]. The single gene encoding TPO is located on chromosome 2p25 containing 17 exons and it spans at least 150 kb [9]. It is a homodimer protein (each monomer consists of 933 amino acid residues) and contains a peroxidase domain, three additional extracellular domains, a transmembrane helix, and a short C-terminal intracellular tail [10]. Although a low-resolution crystal structure of TPO has been reported, its high resolution structure remains to be determined [11, 12]. The closest known homologues of TPO are lactoperoxidase (LPO), myeloperoxidase (MPO), and eosinophil peroxidase (EPO) with a sequence identity of 48%, 47%, and 47%, respectively. X-ray crystallographic structures have been determined for MPO and LPO [13, 14], providing a platform for investigating the structural basis of TPO. Nevertheless, Sarah et al. investigated plausible modes of TPO structure and dimer organization through* in silico* approach [15]. However, how certain mutations affect the TPO protein structure and its enzyme activity is still unknown.

Investigation of mutational spectrum is paramount for genetic disorder study as it gives insight into the disease pathogenicity as well as severity. Screening and identification of mutational spectrum in the TPO gene of patients with TIOD and PIOD have been reported in different countries of the world like Argentina, Netherlands, Japan, Portugal, and China [6, 16–19]. In addition, changes in the TPO enzyme were assayed* in vitro* to compare mutant and wild type activities by several study groups and demonstrated mild to severe TPO enzyme inactivity for some mutations [20, 21]. Though the prevalence of CH is more than twice the global incidence rate in Bangladesh, molecular basis of CH is still unknown in this country. Moreover, the effects of different mutations in the TPO gene on enzyme activity have not been investigated. In this study, we investigated the spectrum of mutations in TPO gene of patients with TDH and explored the possible effect of these mutations on the structure of TPO protein and TPO's MPO-like domain through* in silico* approach such as homology modelling, molecular docking followed by qunatum mechanics/molecular mechanics (QM/MM) and molecular dynamics (MD) simulation.

## 2. Methods and Materials

### 2.1. Study Participants

A total of 36 confirmed cases of congenital hypothyroid patients with dyshormonogenesis were enrolled at the clinical care settings of National Institute of Nuclear Medicine and Allied Sciences (NINMAS) and department of Endocrinology, Bangabandhu Sheikh Mujib Medical University (BSMMU), Dhaka, for their follow-up examinations. Written informed consent was signed by the parents or guardians of the patients. 3 mL of blood was collected in an EDTA tube from each patient. Ethical clearance was obtained from the ethical committee of BSMMU and University of Dhaka.

### 2.2. DNA Isolation and Polymerase Chain Reaction (PCR) Amplification

Genomic DNA was isolated from the EDTA blood by using a Qiagen DNAeasy mini kit. The isolated DNA was then amplified by PCR using TPO gene specific primers that together covered from Exon 8 to Exon 14, since global data showed that most of the common mutations in the TPO gene of the patients with congenital hypothyroidism were confined in this region. The primer sequences are listed in Table 1.

To amplify the desired target sequence of TPO gene, PCR amplification was conducted on a thermal cycler (Bio-Rad, USA). The final reaction volume was 10 *µ*l for each of the reactions which contained 1 *µ*L 10X PCR buffer, 0.3 *µ*L 25 mM MgCl_2_, 2 *µ*L 5X Q-solution, 1.6 *µ*L 2.5 mM dNTPs mixture, 0.2 *µ*L 10 mM Forward and 0.2 *µ*L Reverse primers, 0.05 *µ*L Taq DNA Polymerase, and 50 ng of genomic DNA, and total reaction volume was made up to 10 *µ*L by addition of nuclease free water. The thermal cycling condition included (a) initial denaturation at 95°C for 5 minutes, (b) 35 cycles of denaturation at 95°C for 40 seconds annealing at 58°C for 35 seconds and extension at 72°C for 40 seconds, and (c) final extension at 72°C for 5 minutes.

### 2.3. Sanger Sequencing of PCR Products

Prior to sequencing, PCR product was purified using a Qiagen PCR purification kit (Qiagen) according to manufacturer's instruction. The cycle sequencing PCR was then conducted using a BigDye Chain Terminator version 3.1 Cycle Sequencing Kit (Applied Biosystems, USA) following manufacturer's instructions. The thermal cycling profile included (a) initial denaturation at 94°C for 1 minute, (b) 25 cycles of denaturation at 94°C for 10 seconds, annealing at 58°C for 5 seconds and extension at 60°C for 4 minutes, and (c) final extension at 60°C for 10 minutes. Following completion of cycle sequencing PCR, purification of the product was performed using a BigDye XTerminator® Purification Kit (Applied Biosystems). Finally, sequencing of the purified cycle sequencing product was performed on the ABI PRISM 310 automated sequencer (Applied Biosystems, USA).

### 2.4. Sequencing Data Analysis

Sequencing data were collected using ABI PRISM 310 data collection software version 3.1.0 (Applied Biosystems). Collected FASTA format of sequencing data was used to identify substitution or deletion mutations in the* TPO* gene by alignment with the reference sequence (Accession number; NC_000002.12 retrieved from the NCBI database) using the basic local alignment search tool (BLAST). ExPASy translate tool was used to convert nucleotides sequence into corresponding amino acids.

### 2.5. Analysis of Effect of Three Nonsynonymous Mutations in the 3D Structure of TPO Protein

#### 2.5.1. Prediction of 3D Structure through* In Silico* Approach

We found 4 mutations including three nonsynonymous mutations, namely, p.Ala373Ser; p.Ser398Thr and p.Thr725Pro and a synonymous mutation p.Pro725Pro in the TPO_1-933_ gene. One of our primary goals was to investigate the effect of the nonsynonymous mutations on the 3D structure of MPO-like domain of the TPO protein. In addition, we aimed at seeing whether the mutations had any effects on the heme interactions with specific amino acid residues. The corresponding positions of the mutations, including p.Ala373Ser, p.Ser398Thr, and p.Thr725Pro in the MPO-like domain of TPO_142-738_ were p.Ala232Ser, p.Ser257Thr, and p.Thr584Pro, designated as TPO_142-738_MT1, TPO_142-738_MT2, and TPO_142-738_MT3, respectively. Since the crystallographic structure of TPO protein was available at lower resolution, to understand the effect of mutations in the structure of MPO-like domain of TPO_142-738_, amino acid sequences of wild type and mutant proteins containing different nonsynonymous mutations were submitted to I-TASSER server in order to obtain the 3D structures [22–24]. We obtained 1 model for each structure based on C-score, Template Modelling (TM) score, and Root Mean Square Deviation (RMSD) score. In addition, we investigated the effects of the mutations on full-length TPO protein structure and functions in order to compare with the results with MPO-like domain to see if any changes were found. For this purpose, we also predicted the structures of whole TPO protein (TPO_1-933_) for the wild type (TPO WT) and the mutants (TPO MT1, TPO MT2, and TPO MT3) by using the I-TASSER server and obtained 5 models for each. From the models, we selected the suitable ones based on the organization of its various domains such as myeloperoxidase- (MPO-) like domain (residues 142–738), complement control protein- (CCP-) like domain (residues 740–795) and epidermal growth factor- (EGF-) like domain (residues 796–846) of the whole protein according to published article [15].

#### 2.5.2. Validation of the 3D Structures

The predicted 3D structures of TPO_142-738_ WT, TPO_142-738_MT1, TPO_142-738_ MT2, TPO_142-738_ MT3, TPO_1-933_WT, TPO_1-933_MT1, TPO_1-933_MT2, and TPO_1-933_MT3 were validated using 2 different web servers, namely, Verify3D server, and RAMPAGE server [25–27]. To validate the structure, the PDB format of the predicted 3D structure was submitted to both the servers. The result included the percentage of the amino acids having the average 3D-1D score of ≥ 0.2 for Verify3D server. The RAMPAGE webserver performs Ramachandran Plot Analysis by providing results including the percentages of the amino acid residues within the favored, allowed, and outlier regions. The results of the Verify3D and the RAMPAGE webservers were summarized in the results section.

#### 2.5.3. Optimization of Heme Using Quantum Mechanical (QM) Calculations

Since TPO is a heme containing protein, we investigated the effect of mutation on binding affinity of heme with TPO and its interactions with specific amino acid residues. The initial structure of heme was obtained from the Protein Data Bank (PDB) database, (PDB ID: HEM). Quantum mechanics (QM) calculation had been used to optimize the heme. The QM calculation was conducted using density functional theory (DFT) method employing Becke's (B) exchange functional combining Lee, Yang, and Parr's (LYP) correlation functional, widely known as B3LYP density functional in Gaussian 09 program package [28–30]. SDD basis set had been used for optimization of the heme [31, 32].

#### 2.5.4. Molecular Docking of Heme with the Predicted 3D Structures of TPO

For molecular docking, Autodock Vina [33, 34] protocol was employed. The molecular docking approach involved the prediction of the interaction between a small molecule and a protein at the atomic level, which provided us the opportunity to investigate the behavior of small molecules in the binding site of target proteins and to elucidate the fundamental biochemical processes. Before docking processes, knowing the location of ligand binding sites in the target protein could significantly increase the docking efficiency [35]. The binding sites of heme with TPO had been identified. It was found that Asp238, His239, Arg396, Glu399, and His494 were present in the active site of TPO_1-933_; i.e. MPO-like domain and these amino acid residues were involved with heme interactions and thus the catalytic activity of TPO [36, 37]. But in the case of MPO-like domain of TPO_142-738_, the corresponding position of Asp238, His239, Arg396, Glu399, and His494 would be Asp97, His98, Arg255, Glu258, and His353, respectively. Optimal confined search space had been selected for successful flexible molecular docking of heme with TPO_142-738_ WT, TPO_142-738_ MT1, TPO_142-738_ MT2, and TPO_142-738_ MT3. The center of the grid box for TPO_142-738_ WT was set to 71.337 Å, 73.748 Å, and 72.888 Å; whereas they were set to 71.281 Å, 73.813 Å, and 73.314 Å for TPO_142-738_ MT1; 73.115 Å, 74.888 Å, and 73.156 Å for TPO_142-738_ MT2; and 73.273 Å, 75.433 Å, and 74.247 Å for TPO_142-738_ MT3 in the x, y, and z directions, respectively. The grid box size was set to 25.0 Å, 25.0 Å, and 25.0 Å for TPO_142-738_WT, MT1, MT2, and MT3 in the x, y, and z directions, respectively. Grid box value center and grid box size was also optimized for full-length TPO_1-933_ (Table 2).

#### 2.5.5. Visualization and Analysis of Docking Results

The molecular docking results of heme with TPO_142-738_ WT, TPO_142-738_ MT1, TPO_142-738_ MT2, and TPO_142-738_ MT3 and also for the whole protein TPO_1-933_ were visualized and analyzed using the PyMol (version 2.0) and BIOVIA Discovery Studio 2017 software [38, 39]. The binding affinities of heme with wild type TPO (TPO WT) compared to 3 mutant proteins (TPO MT1, TPO MT2, and TPO MT3) were observed. Moreover, the corresponding non-bond interactions of amino acids with heme were also studied for wild type and mutant 3D structures.

#### 2.5.6. QM/MM and Molecular Dynamics (MD) Simulation

To validate the molecular docking results, further analysis by quantum chemical method QM/MM and molecular dynamics (MD) simulations were employed for full-length TPO proteins (wild type, MT1, and MT3) since published data showed MT1 and MT3 had severe effect on enzyme activity than MT2. The QM/MM calculations of the selected protein-ligand complexes were performed by a two-layer ONIOM method available in the Gaussian09 software package [40–46], QM and MM regions have been shown in supplementary Figure 3. The heme molecule was included in the QM region and semiempirical PM6 level of theory [47] was considered due to large structure of the heme molecules. The regions of full-length protein were computed in the MM region and the Universal Force Field (UFF) was used for the energy minimization. The total ONIOM energy of the entire system is as follows: (see [48])

(1) $$\begin{array}{c} E^{\text{ONIOM}}=E^{\text{real,MM}}+E^{\text{model,QM}}–E^{\text{model,MM}} \end{array}$$

The* real* system consists of all the atoms and is calculated only at the MM level. The* model *system consists of the part of the system (such as heme) that is treated at the QM level [40].

For molecular dynamics simulation, YASARA dynamics program was employed and [48, 49]. AMBER14 force field was considered for all calculations. The size of the cubic simulation box was 167.17 Å*∗* 167.17 Å *∗* 167.17 Å and 151,343 water molecules were added to maintain a solvent density of 1.0 g/ml. The total number of atoms in the system was 469,240. Due to the large size of the protein, we have performed 5000 ps MD simulation [50]. For short-range van der Waals and Coulomb interactions, a cut-off radius of 8.0 Å was considered. Long-range electrostatic interactions were taken into consideration using the Particle Mesh Ewald algorithm [50]. MD simulation was performed for 5000 ps at 310K having a time step of 1.25 fs and the simulation snapshots were saved at every 100 ps. To check whether the ligand stayed in the same binding pocket after 5000 ps of simulation, the heme ligands were retrieved from the simulated ligand-protein complexes and molecular docking was performed again using Autodock Vina protocol as mentioned earlier. The docked structures were analyzed using the same protocol as stated before.

## 3. Result

### 3.1. Demographic Information of the Study Participants

A total of 36 confirmed cases of congenital hypothyroidism with dyshormonogenesis were enrolled in this study. Among 36 patients, 15 (41.67%) and 21 (58.33%) were female and male, respectively, with an average age of 7.58 ± 4.56 years.

### 3.2. Analysis of TPO Gene for Identification of Molecular Basis of Hypothyroidism

As mutations in the TPO gene are commonly associated with thyroid dyshormonogenesis and related complications [18, 51], we opted to analyze the TPO gene to find whether there were mutations in this gene of the study participants. Upon Sanger sequencing of specimens from the patients with thyroid dyshormonogenesis targeting exon-8 to exon-14 which are commonly reported in TPO-associated thyroid dyshormonogenesis, mutations were detected in all 36 samples. A total of four mutations, namely, c.1117G>T (p.Ala373Ser), c.1193G>C (p.Ser398Thr), c.2145C>T (p.Pro715Pro), and c.2173A>C (p.Thr725Pro) were identified in the study participants (Table 3). The first two of these four mutations were detected in exon-8, whereas the remaining two mutations were detected in exon-12. Even though c.2145C>T (or p.Pro715Pro) of the four mutations is a synonymous point mutation and is innocuous, the other three nonsynonymous mutations, namely, p.Ala373Ser, p.Ser398Thr, and p.Thr725Pro, had previously been reported in the patients with thyroid dyshormonogenesis and the reaction kinetics catalyzed by the mutant TPO enzyme proved to be similar with nonenzymatic reaction rates by several other studies [20, 52, 53].

### 3.3. Prediction of 3D Structures of Myeloperoxidase- (MPO-) Like Domain (TPO_142-738_) and Full-Length TPO Protein (TPO_1-933_)

**Figure 1:**
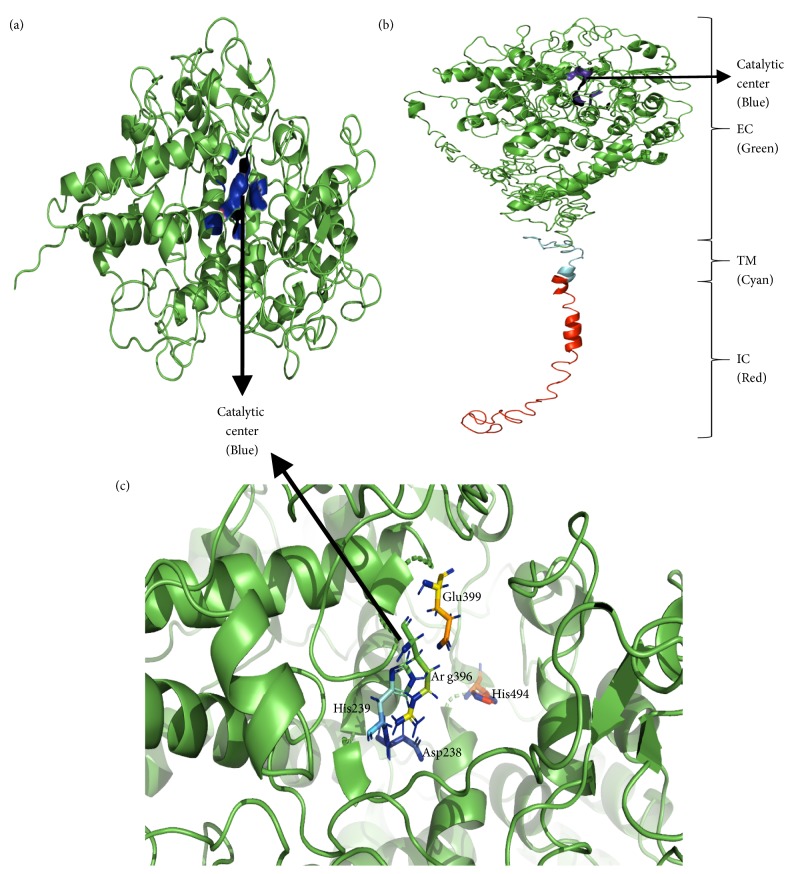
The predicted 3D structures of the proteins. (a) TPO_142-738_ WT, (b) TPO_1-933_ WT, and (c) catalytic site of TPO with crucial amino acids Asp238, His239, Arg396, Glu399, and His494. The specific regions of the TPO_1-933_ WT structure are indicated with the corresponding color written in the brackets. EC = extracellular region shown in green color, TM = transmembrane region shown in cyan color, and IC = intracellular region shown in red color.

The crystallographic structure of TPO protein is available with low resolution and the catalytic domain of TPO_142-738_ is similar to human myeloperoxidase (MPO). Since the mutations that were identified in this present study were confined in the MPO-like domain of TPO protein, we investigated the effect of mutations on the 3D structure of MPO-like domain (TPO_142-738_) of TPO protein. Also, we wanted to see whether the mutations caused any changes in the interactions of various amino acid residues with heme prosthetic group. We submitted the amino acid TPO_142-738_ sequence for the wild type (WT), mutant- MT1, MT2, and MT3 to the I-TASSER server and obtained 3D structures with C-scores of 2, 2, 1.99, and 1.99 for TPO_142-738_ WT, TPO_142-738_ MT1, TPO_142-738_ MT2, and TPO_142-738_ MT3, respectively (Table 4 and Figure 1). To investigate the effects of the mutations on the full-length TPO protein structure and functions, we also predicted the 3D structures for TPO_1-933_ WT, TPO_1-933_ MT1, TPO_1-933_ MT2, and TPO_1-933_ MT3 using the I-TASSER server and compared the results with the MPO-like domain. From the server, we obtained 5 models for each of TPO_1-933_ WT, TPO_1-933_ MT1, TPO_1-933_ MT2, and TPO_1-933_ MT3 and we chose the best model by analyzing the organization of the MPO-like domain (residues 142–738); complement control protein- (CCP-) like domain (residues 740–795); and epidermal growth factor- (EGF-) like domain (residues 796–846) if they were in correct arrangement [54–58] (Figure 1 and Supplementary Figure 2). The corresponding C-score, TM-score, and RMSD score of TPO_1-933_ WT, TPO_1-933_ MT1, TPO_1-933_ MT2, and TPO_1-933_ MT3 were summarized in Table 4.

### 3.4. Validation of the 3D Structures of TPO_142-738_ WT, TPO_142-738_ MT1, TPO_142-738_ MT2, TPO_142-738_ MT3, TPO_1-933_ WT, TPO_1-933_ MT1, TPO_1-933_ MT2, and TPO_1-933_ MT3 Proteins

We validated the 3D structures of TPO WT, MT1, MT2, and MT3 proteins by Verify3D server which measures the accuracy of the predicted 3D structure model with its respective residues (1D) by assigning a structural class based on its location and environment. In Verify3D, more than 80% amino acid residues had the average 3D-1D score of ≥ 0.2 which confers the validity of the 3D structures of TPO_142-738_ WT (96.15%), TPO_142-738_ MT1 (89.78%), TPO_142-738_ MT2 (94.14%), and TPO_142-738_ MT3 (94.64%) (Table 5). Moreover, validation of the structures by the RAMPAGE web server also provided the percentages of the amino acid residues within favored, allowed, and outlier regions. In case of TPO_142-738_ WT, 84.5% residues were within the favored region, 11.6% were within the allowed region, and 3.9% were within the outlier region. For TPO_142-738_ MT1, 84.4% residues were in the favored regions, 11.1% in the allowed region, and 4.4% in the outlier regions. On the one hand, for TPO_142-738_ MT2, 85.2% residues were confined in the favored regions, 10.4% in the allowed region and 4.4% in the outlier regions and on the other hand, for TPO_142-738_ MT3, 81.2% residues were confined in the favored region, 14.3% in the allowed region and 4.5% in the outlier regions (Table 5). The Verify3D results showed that the percentages of amino acid residues having average 3D-1D score of ≥ 0.2 for TPO_1-933_ WT, TPO_1-933_ MT1, TPO_1-933_ MT2, and TPO_1-933_ MT3 were 73.10%, 70.74%, 76.10%, and 76.63%, respectively. The results provided by RAMPAGE server for TPO_1-933_ WT, TPO_1-933_ MT1, TPO_1-933_ MT2, and TPO_1-933_ MT3 were summarized in Table 5.

### 3.5. Optimization of Heme

Density functional theory using B3LYP/SDD was employed for the optimization of the heme prosthetic group. Slight changes in bond distances and bond angles were observed between crystal and DFT structures which are presented in Table 6 and Supplementary Figure 1.

### 3.6. Molecular Docking, Visualization and Analysis of the Docking of Heme with the Predicted Structures of TPO_142-738_ WT, TPO_142-738_ MT1, TPO_142-738_ MT2, TPO_142-738_ MT3, TPO_1-933_ WT, TPO_1-933_ MT1, TPO_1-933_ MT2, and TPO_1-933_ MT3 Proteins

**Figure 2:**
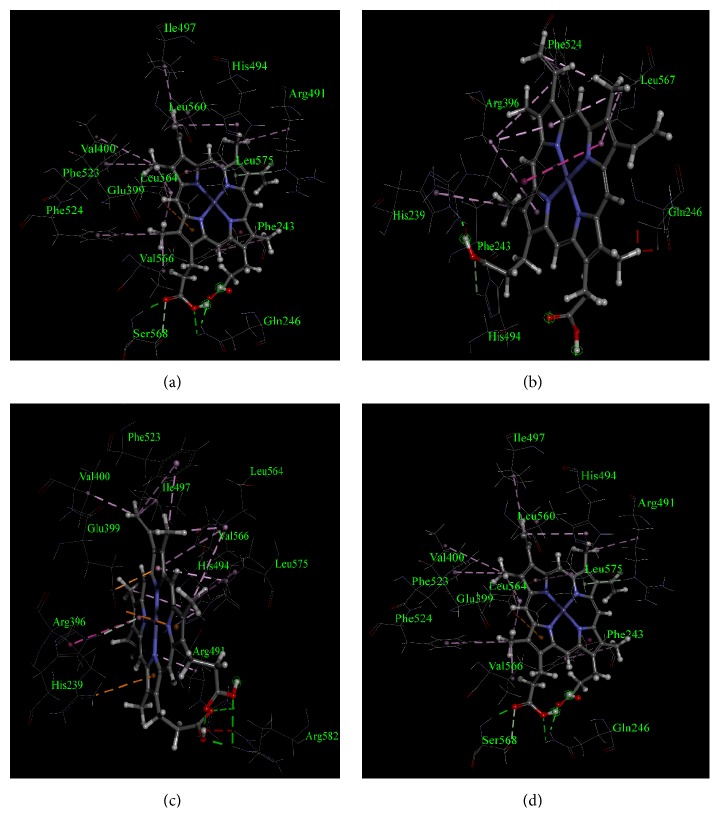
Non-bond interactions of heme with corresponding predicted structures as obtained using a BIOVIA Discovery Studio 2017. (a) TPO_1-933_ WT, (b) TPO_1-933_ MT1, (c) TPO_1-933_ MT2, and (d) TPO_1-933_ MT3. The amino acid residues and their positions are designated as the three letter abbreviations and the corresponding numbers.

**Figure 3:**
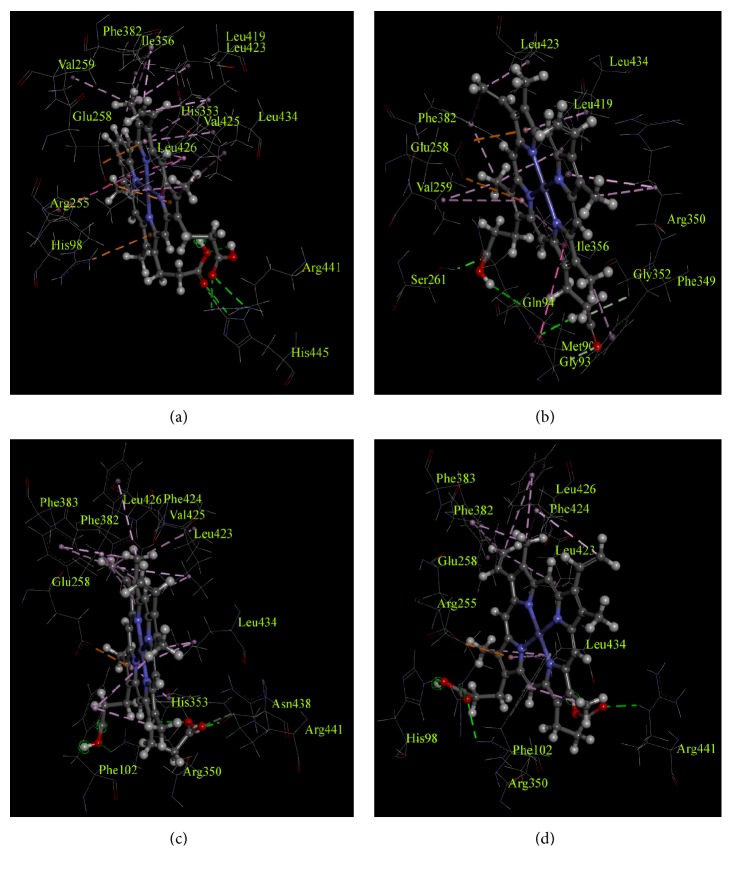
Non-bond interactions of heme with corresponding predicted structures as obtained using a BIOVIA Discovery Studio 2017. (a) TPO_142-738_ WT, (b) TPO_142-738_ MT1, (c) TPO_142-738_ MT2, and (d) TPO_142-738_ MT3. The amino acid residues and their positions are designated as the three letter abbreviations and the corresponding numbers.

From the analysis, it was found that heme interacted with TPO_1-933_ WT through a total of 21 non-bond interactions including interactions with Arg491, and Arg582 through hydrogen bonds, interactions with His239, Val400, Phe490, Arg491, His494, Ile497, Phe523, Leu560, Leu564, and Leu575 through hydrophobic interaction, and interactions with Arg396, Glu399, and Arg491 through electrostatic interactions (Table 7 and Figure 2). Moreover, similar to TPO_1-933_ WT, when MPO-like domain (TPO_142-738_) WT predicted structure was used for docking, 21 non-bond interactions of amino acids with heme were observed as well (Figure 3). However, not all of these 21 interactions for TPO_142-738_ and TPO_1-933_ were common. The TPO_142-738_ WT interactions with heme included Arg582 and Arg586 through hydrogen bonds, His239, Val400, His494, Ile497, Phe523, Leu560, Leu564, Val566, Leu567, and Leu575 through hydrophobic interactions and Arg396, and Glu399 through electrostatic interactions. The common 11 interactions for TPO_1-933_ WT and TPO_142-738_ WT included His239, Arg396, Glu399, Val400, His494, Ile497, Phe523, Leu560, Leu564, Leu575, and Arg582. Moreover, of the aforementioned 11 residues, 4 residues including His239, Arg396, Glu399, and His494 had been reported to correspond to amino acid residues in MPO protein which were crucial for enzyme activity [36, 37].

For TPO_1-933_ MT1, a total of 12 residues were found to interact with heme. Among those residues, 2 residues, namely, His239 and His494, interacted with heme through hydrogen bonds and 5 residues including His239, Phe243, Arg396, Phe524, and Leu567 interacted through hydrophobic interactions (Table 7, Figure 2 and Supplementary Table 2). Total number of interactions decreased significantly for TPO_1-933_ MT1 compared to the TPO_1-933_ WT and the Glu399 residue which is crucial for interaction was also absent in the TPO_1-933_ MT1. On the other hand, when TPO_142-738_ MT1 predicted structure was used for molecular docking, a total of 19 residues were found to interact with heme (Table 7, Figure 3 and Supplementary Table 1). Among those 19 residues, 4 residues including Met231, Gly234, Ser402, and Gly493 interacted with heme through hydrogen bonds, the residues Gly234, Gln235, Val400, Phe490, Arg491, Ile497, Phe523, Leu560, Leu564, and Leu575 interacted through hydrophobic interactions, and the residue Glu399 interacted through electrostatic interaction with the heme (Table 7). However, the crucial interactions of heme with His239, Arg396, and His494 were absent in the TPO_142-738_ MT1. The structure-based docking of both TPO_1-933_ and TPO_142-738_ suggested that MT1 mutation was damaging for the TPO enzyme activity.

For TPO_1-933_ MT2, a total of 20 amino acid residues were found to interact with the heme. Among those residues, 3 residues including His239, Arg491, and Arg582 interacted with heme through hydrogen bonds, 9 residues including His239, Val400, Arg491, His494, Ile497, Phe523, Leu564, Val566, and Leu575 interacted through hydrophobic interactions, and 2 residues including Arg396 and Glu399 interacted through electrostatic interactions (Table 7, Figure 2 and Supplementary Table 2). All four crucial interacting residues, namely, His239, Arg396, Glu399, and His494, which are important for MPO-like domain activity in TPO enzyme, were found to interact with heme in TPO_1-933_ MT2. On the other hand, when TPO_142-738_ MT2 predicted structure was used for docking, a total of 20 residues were found to interact with heme. Among those 20 residues, 3 residues including Arg491, Asn579, and Arg582 interacted with heme through hydrogen bonds, the residues Phe243, His494, Phe523, Phe524, Leu564, Phe565, Val566, Leu567, and Leu575 interacted through hydrophobic interactions and the residue Glu399 interacted through electrostatic interaction (Table 7, Figure 3 and Supplementary Table 1). However, the crucial interactions of heme with residues His239 and Arg396 were absent in the TPO_142-738_ MT2. The TPO_1-933_ structure-based docking showed that the mutations did not result in any major changes in interactions and all major interacting residues were present, whereas the TPO_142-738_ structure-based docking suggested result which was similar to TPO_1-933_ except that the major interacting residues His239 and Arg396 were absent. Together, the docking results are indicative of the fact that this mutation might have an association with PIOD.

For TPO_1-933_ MT3, a total of 21 residues were found to interact with the heme. Among those residues, 3 residues including Gln246, Arg491, and Ser568 interacted with heme through hydrogen bonds, 11 residues including Phe243, Val400, Arg491, His494, Ile497, Phe523, Phe524, Leu560, Leu564, Val566, and Leu575 interacted through hydrophobic interactions, and the residue Glu399 interacted through electrostatic interaction (Table 7, Figure 2 and Supplementary Table 2). Two crucial interacting residues, namely, His239 and Arg396 which are important for MPO-like domain activity in the TPO enzyme, were absent in TPO_1-933_ MT3. On the other hand, when TPO_142-738_ MT3 predicted structure was used for docking, a total of 16 residues were found to interact with heme. Among those 16 residues, 4 residues including His239, Arg396, Arg491, and Arg582 interacted with heme through hydrogen bonds, the residues Phe243, Phe523, Phe524, Leu564, Phe565, Leu567, and Leu575 interacted through hydrophobic interactions and the residue Glu399 interacted through electrostatic interaction (Table 7, Figure 3 and Supplementary Table 1). However, the crucial interactions of heme with His494 were absent in the TPO_142-738_ MT3. Both TPO_1-933_ and TPO_142-738_ structure-based docking with heme suggested that MT3 mutation might have damaging effect to the TPO protein activity.

### 3.7. QM/MM and Molecular Dynamics (MD) Simulation

**Figure 4:**
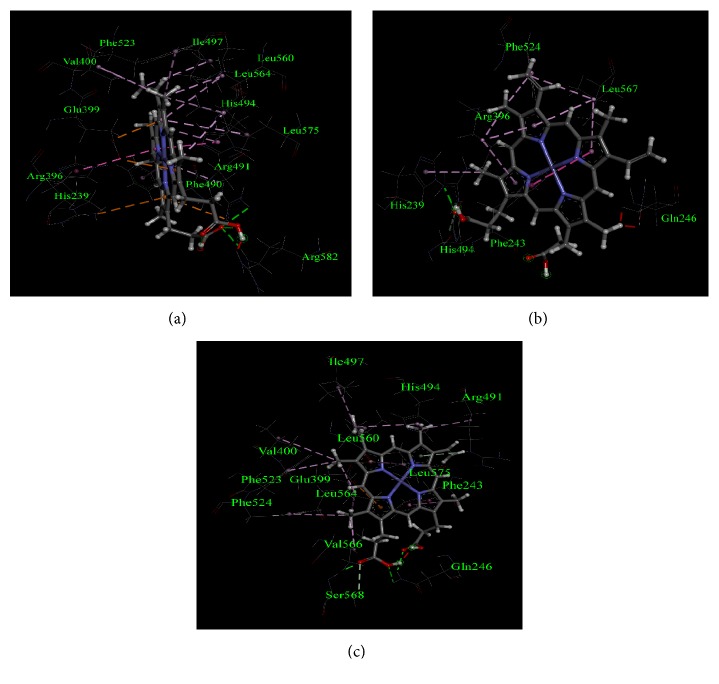
Non-bond interactions of heme with corresponding predicted structures as obtained using a BIOVIA Discovery Studio 2017, after QM/MM calculations of the structures with heme molecule in the active site performed at the ONIOM- (PM6: UFF) level of theory. (a) TPO_1-933_ WT, (b) TPO_1-933_ MT1, and (c) TPO_1-933_ MT3. The amino acid residues and their positions are designated as the three letter abbreviations and the corresponding numbers.

According to Guria et al. TPO_1-933_ MT1 (p.Ala373Ser) and TPO_1-933_ MT3 (p.Thr725Pro) showed more damaging effect on the catalytic activity of TPO protein [20] and our molecular docking-based study showed that full-length TPO_1-933_ MT2 (p.Ser398Thr) structure interacted to all the crucial amino acids in the catalytic site of TPO; thus we selected these TPO_1-933_ MT1 (p.Ala373Ser) and TPO_1-933_ MT3 (p.Thr725Pro) mutant cases for further analysis to validate the molecular docking results and to compare the amino acid interactions with the wild type structure. The interactions between the amino acid residues of TPO_1-933_ WT, TPO_1-933_ MT1, and TPO_1-933_ MT3 proteins and the heme groups were further studied by QM/MM and the mode of interactions is depicted in Figure 4. It was observed that the interacting residues were the same as obtained from the initial docking results.

**Figure 5:**
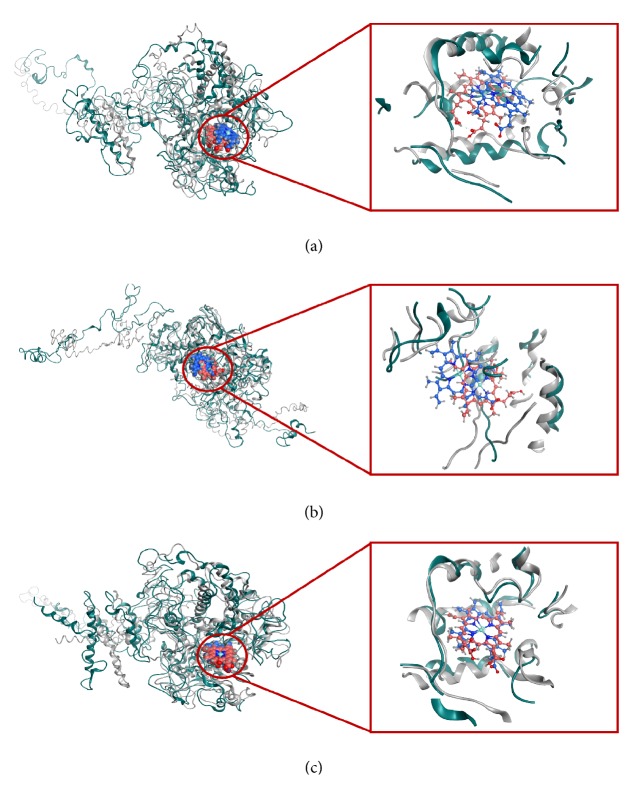
Superimposed structures of protein-ligand (heme) complexes before and after 5000 ps MD simulation. (a) TPO_1-933_ WT, (b) TPO_1-933_ MT1, and (c) TPO_1-933_ MT3. Grey and blue colors indicate results before MD simulation whereas green and pink indicate results after MD simulation.

To investigate the structural changes during molecular dynamics simulation, the protein-ligand complex after 5000 ps of simulation was superimposed on the initial docked protein-ligand complex. The superimposed structures of TPO_1-933_ WT, TPO_1-933_ MT1 (p.Ala373Ser), and TPO_1-933_ MT3 (p.Thr725Pro) proteins are depicted in Figure 5. It was observed that the heme ligand was found within the catalytic sites.

**Figure 6:**
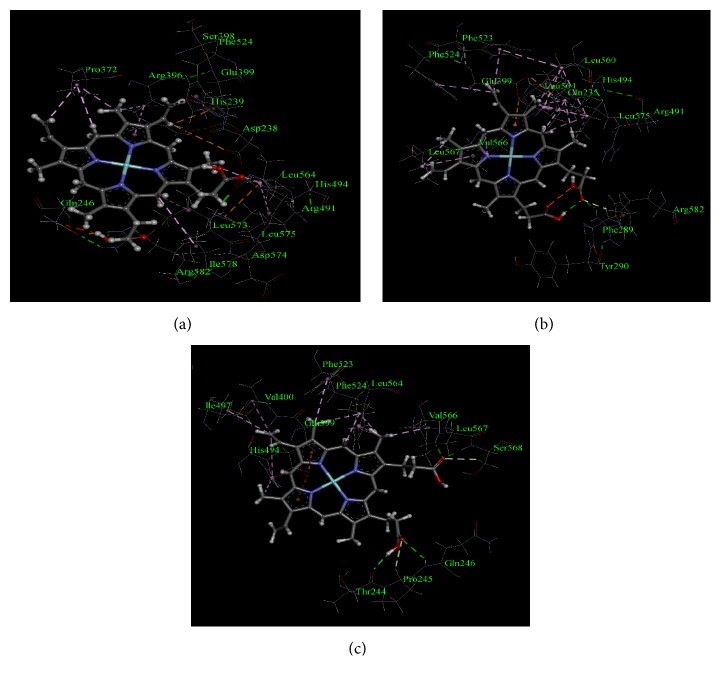
Non-bond interactions of heme with corresponding predicted structures as obtained after docking with the structures retrieved from the 5000 ps simulation using a BIOVIA Discovery Studio 2017. (a) TPO_1-933_ WT, (b) TPO_1-933_ MT1, and (c) TPO_1-933_ MT3. The amino acid residues and their positions are designated as the three letter abbreviations and the corresponding numbers.

As protein flexibility can give rise to difference in binding interactions of a ligand with its target protein, the retrieved protein structure after 5000 ps simulation was again docked with the heme ligand and the results are depicted in Figure 6. From the analysis of molecular docking after performing molecular dynamics of the protein structures, we observed that although heme interacted with TPO_1-933_ WT through all the crucial amino acids (Asp238, His239, Arg396, Glu399, and His494), while it interacted with TPO_1-933_ MT1 and TPO_1-933_ MT3 through Glu399 and His494 residues only. Thus in the mutant cases, there were no interactions for the other 3 amino acid residues, namely, Asp238, His239, and Arg396. As these 5 amino acid residues are important for the catalytic activity of the protein, the absence of interactions with one of these crucial amino acids could affect the functional activity of the protein.

## 4. Discussion

Though congenital hypothyroidism (CH) is the most common preventable disorder, newborn screening is not a regular practice in Bangladesh. Due to late initiation of treatment, many late-diagnosed hypothyroid patients experience various typical signs and symptoms of hypothyroidism even though they receive regular levothyroxine treatment. Although several genes have been reported to be involved in thyroid dyshormonogenesis (TDH), mutation in the TPO gene is frequently described with mild to severe repercussions resulting in partial iodine organification defect (PIOD) to total iodine organification defect (TIOD) [21]. In this present study, we investigated (a) the mutational spectrum in the TPO gene of TDH patients and (b) the influence of specific mutation on TPO protein structure by means of* in silico* approach. To the best of our knowledge, this is the first molecular investigation on genetic etiology of TDH in Bangladesh.

In this study, we analyzed exons 8-14 of TPO gene of the TDH patients as the previous studies had reported that this region was crucial for the enzymatic activity and mutations in this region may result in absence or reduction in TPO activity [20, 59]. Analysis of 36 specimens revealed three nonsynonymous mutations including p.Ala373Ser (TPO MT1), p.Ser398Thr (TPO MT2), and p.Thr725Pro (TPO MT3) and one synonymous mutation p.Pro715Pro. The identified nonsynonymous mutations had previously been reported to be pathogenic or disease-causing mutations [51, 53]. Moreover, a cloning-based study involving aforementioned mutations by Guria et al. reported that these mutations could result in low expression of TPO mRNAs as well as a reduction in TPO enzyme activity [20]. They also demonstrated that mutation p.Ala373Ser (TPO MT1) was more damaging than mutation p.Ser398Thr (TPO MT2). This phenomenon could be explained by a change in aliphatic amino acid to hydroxyl-containing amino acid at 373^th^ position of TPO protein, whereas the other mutation (p.Ser398Thr) did not result in such a shift from one group of amino acids to another. However, the mutation in exon-12, namely, p.Thr725Pro (TPO MT3), could result in a failure of TPO protein to shift to its active state as it is well reported that threonine is the phosphorylation site for protein activation [60, 61]. Moreover, p.Thr725Pro had been reported to be associated with autoimmune hypothyroidism [62]. As CH is genetically and phenotypically diverse, molecular studies may provide additional information for diagnosis, classification, and prognosis of the disease. Particularly, in patients with normal thyroid gland morphology, it could be difficult to distinguish between thyrotropin resistance and dyshormonogenesis; and molecular genetic studies may reveal true etiology of the disease in these cases. In this study, at least one of this three damaging mutations was found in a homozygous state or two of them were in a heterozygous state in each of the study participants. We previously reported a mutational hot-spot in the HBB gene and established a cost-effective molecular method (high resolution melting curve analysis) for screening of HBB gene mutations in Bangladesh [63]. Such a cost-effective approach can be adopted for TDH patients and carrier screening targeting the TPO genes in Bangladesh.

Substitution of an amino acid affects the shape, function, or binding properties of a given protein. With growing importance of genetics and genomics in health sector, considerable efforts have been devoted to linking human phenotypes to genotypic variations at the nucleotide level and associated changes in 3D protein structure [64, 65]. Since the identified mutations were pathogenic, we wanted to investigate how these mutations were related to dyshormonogenesis by affecting the structural integrity and function of TPO protein. To date, there is no high resolution X-ray crystallographic structure for any of the TPO proteins (wild type or mutated) in the public databases. However, there were structures for closely related proteins, namely, myeloperoxidases (MPO) and lactoperoxidases (LPO) [11, 12]. Thus we predicted and validated the three dimensional (3D) structures of TPO (WT and MT) protein using various bioinformatics tools. We studied the effects of mutations on the structural integrity, arrangement of various domains, and folding patterns of the TPO protein. To further study mutational effects, we performed molecular docking of heme in the catalytic site of TPO to investigate how these mutations could affect the activity of TPO enzyme. We obtained the binding energies of heme for the wild type and the mutant structures of TPO protein and found a reduction in binding affinities in the mutant structures compared to the wild type one. Further, we analyzed the detailed results of molecular docking by observing the non-bond interactions of heme with specific amino acid residues to understand the effects of mutations on the functions of TPO protein.

In our study, we applied our bioinformatics approaches on the MPO-like domain of TPO (TPO_142-738_) and full-length TPO protein (TPO_1-933_) targeting the non-bond interactions between the heme group and amino acid residues as the mutations identified in this study were found in this region and this MPO-like domain was the catalytic site of TPO enzyme [54]. We obtained the predicted structures for the wild type and the mutant proteins from the I-TASSER server for TPO_142-738_ and all the structures had significant confidence score (C-score) suggesting that the structures were valid for further studies [22]. However, I-TASSER predicted structures for TPO_1-933_ had lower C-score value and the reasons may be due to prediction for a very large protein, because I-TASSER predicts structures which are based on iterative threading and also homology modelling and as TPO has no crystallographic structure, this still remains a challenge [22, 24]. We also verified the structures using the Verify3D and the RAMPAGE web servers and both of the servers gave satisfactory results [27, 66]. As heme is crucial for the catalytic activity of TPO, the molecular docking of heme with the wild type and the mutant TPO structures could help us to understand the effects of mutations on the functions of TPO protein. We observed a decrease in heme binding energies in the cases of mutant TPO proteins suggesting that the catalytic activity of mutant TPO might have been hampered. Further investigation gave us information about the heme interactions with specific amino acid residues of TPO protein. The heme prosthetic group of wild type structure interacted with all the important amino acid residues including His239, Arg396, Glu399 and His494 through non-bond interactions. But in the cases of mutant structures, some of the important amino acid interactions were absent, suggesting that the mutations might have damaging effects on heme interactions and thereby affecting the catalytic activity of TPO enzyme. Guria et al. demonstrated that MT1 (p.Ala373Ser), MT2 (p.Ser398Thr), and MT3 (p.Thr725Pro) had damaging effect on TPO mRNA expression and enzyme activity [20]. However, MT1 (p.Ala373Ser) and MT3 (p.Thr725Pro) showed iodination reactions similar to the nonenzymatic reaction rate, suggesting that these two mutations were more damaging than the MT2 (p.Ser398Thr) which showed more efficient iodination reaction than MT1 and MT3 but less efficient than the wild type TPO protein [20]. This finding is consistent with our molecular docking-based study targeting both the MPO-like domain of TPO (TPO_142-738_) and the full-length TPO protein (TPO_1-933_) predicted structures. MT1 and MT3 had more enhanced influence on the interactions between several crucial residues and the heme group, whereas MT2 had less influence on the interaction between the TPO protein and the heme prosthetic group. Different studies showed that quantum chemical methods such as DFT or QM/MM can also be employed for investigating the intermolecular interactions [67–69]. Thus, to validate the results of molecular docking, we performed QM/MM calculations on the full-length wild type TPO protein and two mutant proteins (MT1 and MT3) that had severe damaging effect on the catalytic activity of TPO.

The heme group was treated using a semiempirical approach of calculations and the protein was treated using an approach of molecular mechanical calculations. The structures obtained after performing QM/MM calculations were taken into consideration for further analysis of the presence of non-bond interactions of the active site residues with the heme group. The interactions observed were exactly similar to those obtained from molecular docking, suggesting that the docking results obtained were valid.

In cellular environment, protein structures are always in a dynamic condition. To mimic the cellular environment, we performed molecular dynamics simulation in a cubic simulation box containing water molecules and NaCl and a pH of 7.4 was maintained. After performing 5000 ps simulation under the influence of AMBER14 force field, the final protein structures were superimposed on the corresponding docked protein structure. The results infer that the protein-ligand complexes studied were considerably stable over time as there was very small change in the coordinates of the heme group after the simulation. To observe the effect of protein dynamics, the protein structures were retrieved from the 5000 ps simulation snapshot and again docked with the heme ligand. The finding was rather interesting as the wild type protein was still found to be interacting with the crucial amino acid residues. However, these important interactions were found to be absent for the mutant proteins that might be a potential cause of a decrease in the enzymatic activity of TPO protein.

To understand the in-depth characteristics of TPO protein of both wild type and mutant forms, we performed* in silico* approach to mimic cellular environment. However, various computational chemistry-based approaches like IR and Raman spectroscopy can be used to identify the changes in molecular level with higher specificity [70, 71]. Such compositional analysis in various cellular conditions has become very popular for characterizing the biochemical changes in various disease conditions and also for the study on characterizing the spectrum of various hormones such as corticosteroids [70–72]. The study by Claudio et al. analyzed the molecular vibrational spectrum of thyroid tissues from normal and disease conditions which could ultimately represent the characteristics of secondary structures of proteins [73]. Although use of IR and Raman spectroscopy could offer more insights into the physiological conditions of TDH patients carrying mutations in the TPO gene, the present study was not subjected to such approaches because we did not have such facilities.

In this present study, we had used computational approaches such as molecular docking, QM/MM and molecular dynamics simulation to investigate the effect of mutations on TPO protein interactions. The present study could be useful for future studies including study of the effect of mutations on TPO dimer organization and how mutations in the TPO gene could lead to a change in the TPO protein conformation.

## 5. Conclusion

Our study investigated the genetic etiology of Bangladeshi patients with TDH, which may further help us to screen and categorize the disease. Three pathogenic mutations were observed in the patients with TDH including p.Ala373Ser, p.Ser398Thr, and p.Thr725Pro.* In silico* based study revealed that p.Ala373Ser and p.Thr725Pro mutations resulted in a significant change in interactions between the amino acid residues of TPO protein and the heme group, whereas the p.Ser398Thr mutation could affect the TPO protein to a lesser extent. The results of this study may help better understand the correlation between specific mutation in the TPO gene and altered biological activities of the TPO protein as well as disease severity among the TDH patients. Furthermore, future study on dimer formation of TPO protein and functional activity study of TPO enzyme in physiological condition is expected to shed light on how mutation in the TPO gene can affect thyroid hormone biosynthesis pathway and find a mutation-based better treatment strategy for individual patients.

## Figures and Tables

**Table 1:** List of primers for PCR amplification and Sanger sequencing of TPO gene.

**Primer Name**	**Sequence (5**′**-3**′**)**	**Product size (base pair)**
TPO_Ex8F	TCCAGAGTCTTACAAAGGGTGC	679
TPO_Ex8R	GTACCTGGGAGAGAGAAGCCAC	

TPO_Ex9F	GAGGTGCTGTCTCTTGCCACTG	568
TPO_Ex9R	GGAAGAGTTCATGGGGACCAGC	

TPO_Ex10F	TAGAACTGAGCCAAGAGCTGTC	292
TPO_Ex10R	CTAGCAGCAGGTTGCTAGCTCG	

TPO_Ex11F	GACCATGGCATGAGTGAGATGG	363
TPO_Ex11R	CTGCCTCTGTGCAGAACGTG	

TPO_Ex12F	GGTTCTCCATGCACTGTGACCT	1044
TPO_Ex13R	GATTCCACGTGCCTGTCCTGAG	

TPO_Ex14F	CCTCATCACCTTTTCGGATGTGC	512
TPO_Ex14R	CAGACAGCAGGCACACGAAGTG	

**Table 2:** Grid box center and grid box size for full-length TPO_1-933_ WT, MT1, MT2, and MT3.

Proteins	Grid box center (Å)			Grid box size (Å)		
	X	Y	Z	X	Y	Z
TPO_1-933_	109.7751	128.9632	119.5649	24.5891	20.8773	31.4612
WT						
TPO_1-933_	105.160	102.301	98.7798	25.0	25.0	25.0
MT1						
TPO_1-933_	99.6151	102.226	100.5057	25.0	25.0	25.0
MT2						
TPO_1-933_	102.196	124.345	113.7515	25.0	25.0	25.0
MT3						

**Table 3:** Mutation detection in the TPO gene of hypothyroid patients.

**Sl no.**	**Exon**	**Nucleotide position**	**Amino acid position**	**Functional change (polarity)**	**Reference**
1	8	c.1117G>T	p.Ala373Ser	Similar to non-enzymatic reaction rate	[20, 52]
2	8	c.1193G>C	p.Ser398Thr	Low enzymatic reaction rate	[20, 52]
3	12	c.2145C>T	p.Pro715Pro	Not applicable	[20, 52, 53]
4	12	c.2173A>C	p.Thr725Pro	Similar to non-enzymatic reaction rate	[20, 52, 53]

**Table 4:** Summary of the corresponding model numbers, C-score, TM-score, and the RMSD-score of the predicted 3D structures of TPO_142-738_ WT, TPO_142-738_ MT1, TPO_142-738_ MT2, TPO_142-738_ MT3, TPO_1-933_ WT, TPO_1-933_ MT1, TPO_1-933_ MT2, and TPO_1-933_ MT3.

Features	TPO_142-738_	TPO_142-738_	TPO_142-738_	TPO_142-738_	TPO_1-933_	TPO_1-933_	TPO_1-933_	TPO_1-933_
	WT	MT1	MT2	MT3	WT	MT1	MT2	MT3
Model no.	01	01	01	01	02	01	05	01
C-score	2	2	1.99	1.99	–3.35	–3.23	–3.27	–2.9
TM-score	0.99 ± 0.04	0.99 ± 0.04	0.99 ± 0.04	0.99 ± 0.04	–	0.35 ± 0.12	–	0.38 ± 0.13
RMSD (Å)	3.5 ± 2.4	3.4 ± 2.4	3.6 ± 2.5	3.6 ± 2.5	–	17.2 ± 2.7	–	16.2 ± 3.1

C-score = confidence score range: [-5,2]; TM-score = Template Modelling score, TM-score < 0.17 indicates random similarity and TM-score > 0.5 indicates correct similarity; RMSD = Root Mean Square Deviation. WT = wild type, MT1 = mutant 1 (p.Ala373Ser), MT2 = mutant 2 (p.Ser398Thr), and MT3 = mutant 3 (p.Thr725Pro).

**Table 5:** Summary of the Verify3D and RAMPAGE webserver results for the predicted 3D structures of TPO_142-738_ WT, TPO_142-738_ MT1, TPO_142-738_ MT2, TPO_142-738_ MT3, TPO_1-933_ WT, TPO_1-933_ MT1, TPO_1-933_ MT2, and TPO_1-933_ MT3 proteins.

		TPO_142-738_	TPO_142-738_	TPO_142-738_	TPO_142-738_	TPO_1-933_	TPO_1-933_	TPO_1-933_	TPO_1-933_
		WT	MT1	MT2	MT3	WT	MT1	MT2	MT3
Verify3D		96.15%	89.78%	94.14%	94.64%	73.10%	70.74%	76.10%	76.63%
RAMP-AGE	Favored region	84.5%	84.4%	85.2%	81.2%	68.40%	69.60%	66.90%	69.80%
	Allowed region	11.6%	11.1%	10.4%	14.3%	17.20%	16.20%	18.40%	18.80%
	Outlier region	3.9%	4.4%	4.4%	4.5%	14.40%	14.20%	14.70%	11.40%

Verify3D: percentages of amino acids having the average 3D-1D score ≥ 0.2; RAMPAGE: percentages of the amino acid residues within the favored, allowed, and outlier regions. WT = wild type, MT1 = mutant 1 (p.Ala373Ser), MT2 = mutant 2 (p.Ser398Thr), and MT3 = mutant 3 (p.Thr725Pro).

**Table 6:** Summarized data for the bond distances and bond angles of specific atoms of heme before and after optimization.

X-ray Structure		DFT structure		X-ray Structure		DFT Structure	
Atoms	Bond distance (nm)	Atoms	Bond distance (nm)	Atoms	Bond angle (degrees)	Atoms	Bond angle (degrees)
39N...43Fe	1.977	13N...43Fe	2.013	39N...43Fe...40N	82.23°	13N...43Fe...14N	89.78°
40N...43Fe	2.393	14N...43Fe	2.008	40N...43Fe...41N	80.66°	14N...43Fe...15N	89.97°
41N...43Fe	2.088	15N...43Fe	2.008	41N...43Fe...42N	94.79°	15N...43Fe...42N	90.04°
42N...43Fe	1.845	42N...43Fe	2.011	39N...43Fe...42N	98.39°	42N...43Fe...13N	90.22°

**Table 7:** Binding energy and non-bond interactions of heme with TPO_142-738_ WT, TPO_142-738_ MT1, TPO_142-738_ MT2, TPO_142-738_ MT3, TPO_1-933_ WT, TPO_1-933_ MT1, TPO_1-933_ MT2, and TPO_1-933_ MT3 proteins after flexible docking.

**Protein type**	**Binding Affinity (kcal/mol)**	**Hydrogen bond**	**Hydrophobic bond**	**Electrostatic bond**	**Total interactions**
TPO_142-738_ WT	–11.9	Arg441(Arg582), Arg445(Arg586)	His98(His239), Val259(Val400), His353(His494), Ile356(Ile497), Phe382(Phe523), Leu419(Leu560), Leu423(Leu564), Val425(Val566), Leu426(Leu567), Leu434(Leu575)	Arg255(Arg396), Glu258(Glu399)	21

TPO_142-738_ MT1	–10.8	Met90(Met231), Gly93(Gly234), Ser261(Ser402), Gly352(Gly493)	Gly93(Gly234), Gln94(Gln235), Val259(Val400), Phe349(Phe490), Arg350(Arg491), Ile356(Ile497), Phe382(Phe523), Leu419(Leu560), Leu423(Leu564), Leu434(Leu575)	Glu258(Glu399)	19

TPO_142-738_ MT2	–2.5	Arg350(Arg491), Asn438(Asn579), Arg441(Arg582)	Phe102(Phe243),His 353(His494), Phe382(Phe523), Phe383(Phe524), Leu423(Leu564), Phe424(Phe565), Val425(Val566), Leu426(Leu567), Leu434(Leu575)	Glu258(Glu399)	20

TPO_142-738_ MT3	–5.3	His98(His239), Arg255(Arg396), Arg350(Arg491), Arg441(Arg582)	Phe102(Phe243), Phe382(Phe523), Phe383(Phe524), Leu423(Leu564), Phe424(Phe565), Leu426(Leu567)Leu 434(Leu575)	Glu258(Glu399)	16

TPO_1-933_ WT	–11.5	Arg491, Arg582	His239, Val400, Phe490, Arg491, His494, Ile497, Phe523, Leu560, Leu564, Leu575	Arg396, Glu399, Arg491	21

TPO_1-933_ MT1	–3.2	His239, His494	His239, Phe243, Arg396, Phe524, Leu567	Not found	12

TPO_1-933_ MT2	–11.5	His239, Arg491, Arg582	His239, Val400, Arg491, His494, Ile497, Phe523, Leu564, Val566, Leu575	Arg396, Glu399	20

TPO_1-933_ MT3	–7.9	Gln246, Arg491, Ser568	Phe243, Val400, Arg491, His494, Ile497, Phe523, Phe524, Leu560, Leu564, Val566, Leu575	Glu399	21

The amino acid residues and their positions are designated as the three letter abbreviations and the corresponding number; in case of TPO_142-738_ the amino acid outside the first bracket indicates the position in predicted structure for TPO_142-738_ and the amino acid residues in first bracket indicates the real position in TPO_1-933_ protein; WT = wild type; MT1 = mutant 1 (p.Ala373Ser); MT2 = mutant 2 (p.Ser398Thr); MT3 = mutant 3 (p.Thr725Pro).

## Data Availability

The data used to support the findings of this study are included within the article.

## Supplementary Materials

Supplementary Materials Supplementary Figure 1: Structural differences of heme before and after optimization from three different points of view. Supplementary Figure 2: The predicted 3D structures of the proteins. (A) TPO_1-933_ WT, (B) TPO_1-933_ MT1, (C) TPO_1-933_ MT2, and (D) TPO_1-933_ MT3. The specific regions of the structures are indicated with the corresponding color written in the brackets. EC = extracellular region shown in green color, TM = transmembrane region shown in cyan color, and IC = intracellular region shown in red color, catalytic center shown in blue color. Supplementary Figure 3: QM/MM ONIOM calculation set up for protein-ligand complexes, high level (ball and stick), and low level (line). Supplementary Table 1: Binding energies and non-bond interactions of heme with TPO_142-738_ WT, TPO_142-738_ MT1, TPO_142-738_ MT2, and TPO_142-738_ MT3 after flexible docking. Supplementary Table 2: Binding energies and non-bond interactions of heme with TPO_1-933_WT, TPO_1-933_MT1, TPO_1-933_ MT2, and TPO_1-933_ MT3 after flexible docking.
