# Supplementary material for: Mutation Spectrum in TPO Gene of Bangladeshi Patients with Thyroid Dyshormonogenesis and Analysis of the Effects of Different Mutations on the Structural Features and Functions of TPO Protein through In Silico Approach
Source: Biomed Res Int. 2019 Feb 24;2019:9218903. doi: 10.1155/2019/9218903 (PMC6409061; doi:10.1155/2019/9218903)
Supplement: Supplementary Materials — Supplementary Figure 1: Structural differences of heme before and after optimization from three different points of view. Supplementary Figure 2: The predicted 3D structures of the proteins. (A) TPO1-933 WT, (B) TPO1-933 MT1, (C) TPO1-933 MT2, and (D) TPO1-933 MT3. The specific regions of the structures are indicated with the corresponding color written in the brackets. EC = extracellular region shown in green color, TM = transmembrane region shown in cyan color, and IC = intracellular region shown in red color, catalytic center shown in blue color. Supplementary Figure 3: QM/MM ONIOM calculation set up for protein-ligand complexes, high level (ball and stick), and low level (line). Supplementary Table 1: Binding energies and non-bond interactions of heme with TPO142-738 WT, TPO142-738 MT1, TPO142-738 MT2, and TPO142-738 MT3 after flexible docking. Supplementary Table 2: Binding energies and non-bond interactions of heme with TPO1-933WT, TPO1-933MT1, TPO1-933 MT2, and TPO1-933 MT3 after flexible docking. [file 9218903.f1.pdf]

Supplementary information

**A) Before optimization**	**B) After optimization**
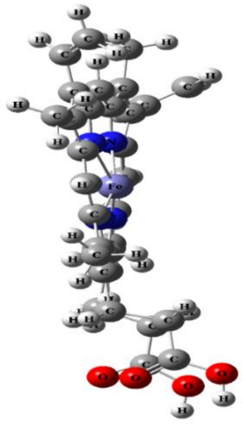 View 1	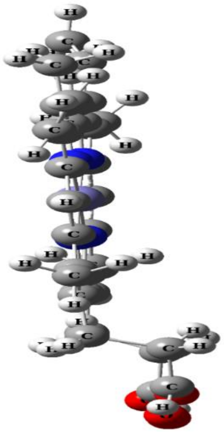 View 1
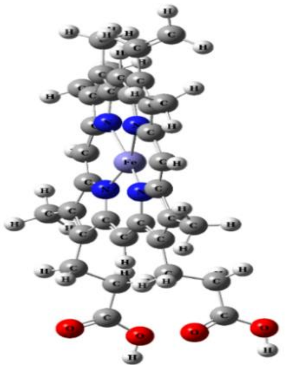 View 2	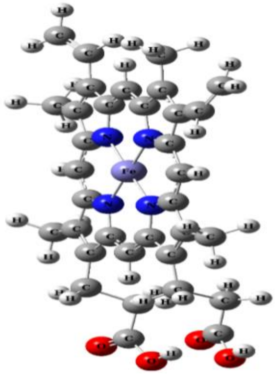 View 2
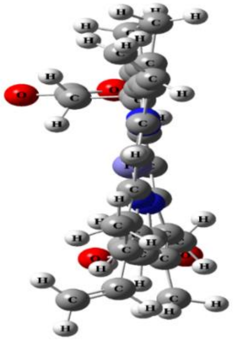 View 3	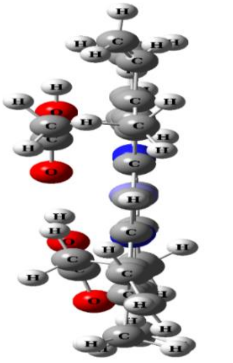 View 3

Supplementary Figure 1: Structural differences of heme before and after optimization from three different points of view.

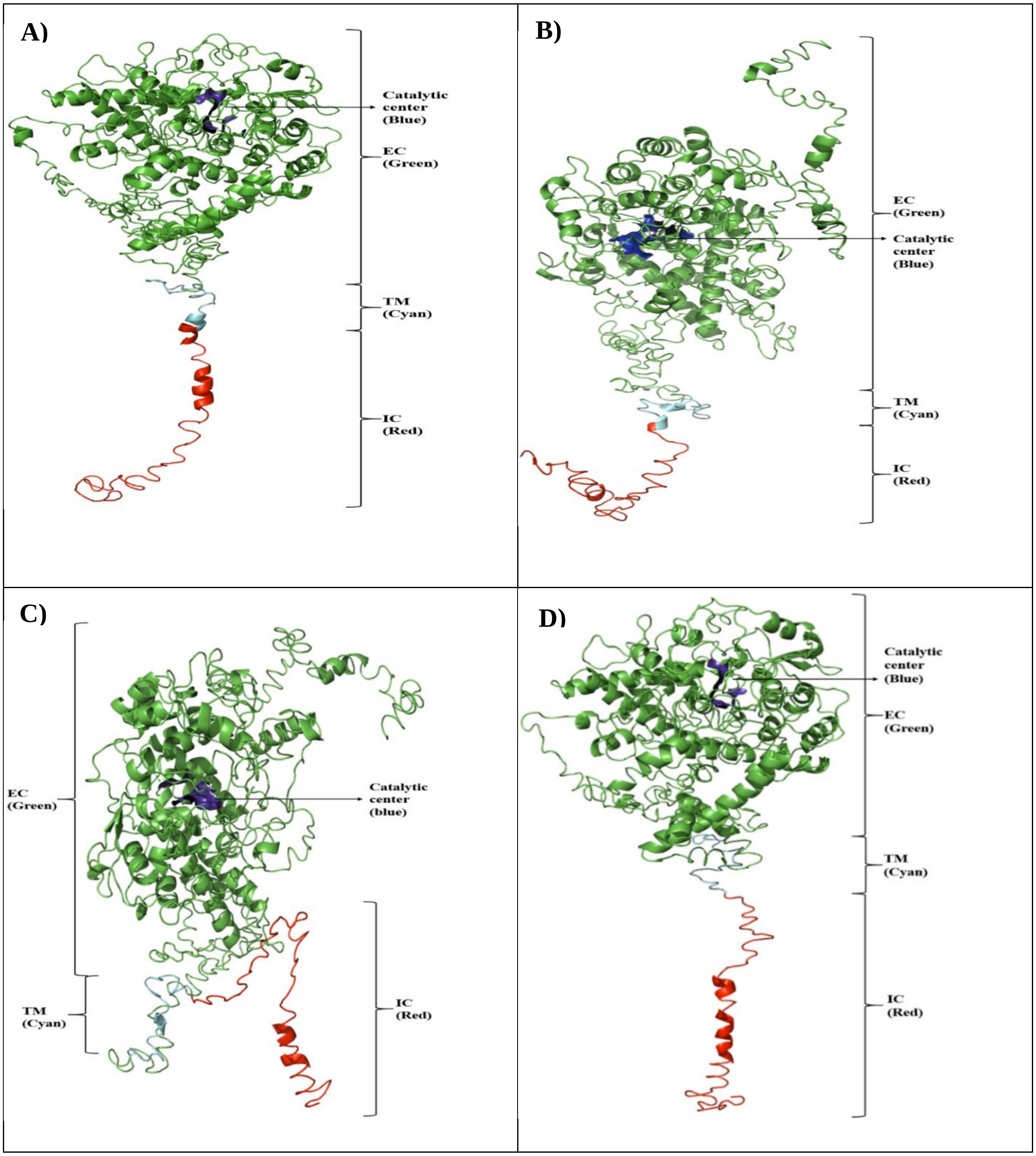

Supplementary Figure 2: The predicted 3D structures of the proteins. (A) TPO_{1-933} WT, (B) TPO_{1-933} MT1, (C) TPO_{1-933} MT2, (D) TPO_{1-933} MT3. The specific regions of the structures are indicated with the corresponding color written in the brackets. EC=Extracellular region shown in green

color, TM=Transmembrane region shown in cyan color, IC=Intracellular region shown in red color, Catalytic center shown in blue color.

Supplementary Table 1. Binding energies and non-bond interactions of Heme with TPO$_{142-738}$ WT, TPO$_{142-738}$ MT1, TPO$_{142-738}$ MT2, TPO$_{142-738}$ MT3 after flexible docking.

Protein	Binding energy (kcal / mol)	Category	Interacting amino acids	Interaction types	Distance (Å)	Total no. of interactions
TPO$_{142-738}$ WT	–11.9	Hydrogen bond	Arg441(Arg582)	Conventional Hydrogen bond	2.668	21
					2.897	
					2.489	
			Arg445(Arg586)	Carbon Hydrogen bond	2.983	
		Hydrophobic interactions	His98(His239)	Pi-Pi T-Shaped	5.555	
			Val259(Val400)	Alkyl	4.305	
			His353(His 494)	Pi-Pi T-Shaped	4.356	
				Pi-Alkyl	5.235	
					4.876	
			Ile356(Ile497)	Alkyl	4.854	
			Phe382(Phe523)	Pi-Alkyl	5.023	
					4.113	
			Leu419(Leu560)	Alkyl	5.171	
			Leu423(Leu564)	Alkyl	4.517	
				Pi-Alkyl	5.288	
			Val425(Val566)	Alkyl	4.845	
			Leu426(Leu567)	Alkyl	5.458	
			Leu434(Leu575)	Pi-Alkyl	5.089	
		Electrostatic interactions	Arg255(Arg396)	Pi-Cation	4.461	
			Glu258(Glu399)	Pi-Anion	4.296	
					3.602	
TPO$_{142-738}$ MT1	–10.8	Hydrogen bond	Met90(Met231)	Conventional Hydrogen bond	2.327	19
					2.938	
			Gly93(Gly234)	Carbon Hydrogen bond	2.338	
			Ser261(Ser402)	Conventional Hydrogen bond	2.109	

			Gly352(Gly493)	Carbon Hydrogen bond	2.792	
		Hydrophobic interactions	Gly93(Gly234)	Amide Pi Stacked	5.0	
			Gln94(Gln235)			
			Val259(Val400)	Alkyl	3.566	
				Pi-Alkyl	5.471	
			Phe349(Phe490)	Pi-Alkyl	4.054	
			Arg350(Arg491)	Alkyl	4.666	
				Pi-Alkyl	5.031	
			Ile356(Ile497)	Pi-Alkyl	5.193	
			Phe382(Phe523)	Pi-Alkyl	3.95	
					4.787	
			Leu419(Leu560)	Alkyl	5.252	
			Leu423(Leu564)	Alkyl	4.464	
			Leu434(Leu575)	Pi-Alkyl	4.935	
		Electrostatic interactions	Glu258(Glu399)	Pi-Anion	3.914	
					4.651	
$TPO_{142-738}$ MT2	–2.5	Hydrogen bond	Arg350(Arg491)	Conventional Hydrogen bond	2.598	20
					2.96	
			Asn438(Asn579)		2.038	
			Arg441(Arg582)	Carbon Hydrogen bond	2.937	
		Hydrophobic interactions	Phe102(Phe243)	Pi-Alkyl	5.333	
					4.615	
			His353(His494)	Pi-Alkyl	4.462	
			Phe382(Phe523)	Pi-Alkyl	3.678	
			Phe383(Phe524)	Pi-Alkyl	4.897	
					4.558	
			Leu423(Leu564)	Alkyl	5.269	
				Pi-Alkyl	5.159	
			Phe424(Phe565)	Pi-Alkyl	5.105	
			Val425(Val566)	Alkyl	4.147	
			Leu426(Leu567)	Alkyl	3.903	
					3.824	
				Pi-Alkyl	5.226	
			Leu434(Leu575)	Alkyl	5.139	
					5.238	
		Electrostatic interactions	Glu258(Glu399)	Pi-Anion	3.338	
$TPO_{142-738}$ MT3	–5.3	Hydrogen bond	His98(His239)	Conventional Hydrogen bond	1.967	16
			Arg255(Arg396)		2.793	
			Arg350(Arg491)		2.686	
			Arg441(Arg582)		2.36	

		Hydrophobic interactions	Phe102(Phe243)	Pi-Alkyl	4.516	
			Phe382(Phe523)	Pi-Alkyl	3.675	
			Phe383(Phe524)	Pi-Alkyl	3.622	
					4.795	
			Leu423(Leu564)	Alkyl	4.628	
			Phe424(Phe565)	Pi-Alkyl	4.705	
					5.304	
			Leu426(Leu567)	Alkyl	4.406	
					4.555	
			Leu434(Leu575)	Alkyl	4.991	
				Pi-Alkyl	5.148	
		Electrostatic interactions	Glu258(Glu399)	Pi-Anion	3.633	

The amino acid residues and their positions are designated as the three letter abbreviations and the corresponding number; in case of TPO$_{142-738}$ the amino acid outside the first bracket indicates the position in predicted structure for TPO$_{142-738}$ and the amino acid residues in first bracket indicates the real position in TPO$_{1-933}$ protein; WT = Wild type; MT1 = Mutant 1 (p.Ala373Ser); MT2 = Mutant 2 (p.Ser398Thr); MT3 = Mutant 3 (p.Thr725Pro).

Supplementary Table 2: Binding energies and nonbond interactions of Heme with TPO$_{1-933}$**WT,** TPO$_{1-933}$**MT1,** TPO$_{1-933}$ **MT2 and** TPO$_{1-933}$ **MT3 after flexible docking.**

Protein	Binding energy (kcal / mol)	Category	Interacting amino acids	Interaction types	Distance (Å)	Total no. of interactions
TPO$_{1-933}$ WT	–11.5	Hydrogen bond	Arg491	Conventional Hydrogen bond	2.579	21
					2.765	
			Arg582		2.445	
		Hydrophobic interactions	His239	Pi-Pi T-Shaped	5.589	
			Val400	Alkyl	4.169	
					4.851	
			Phe490	Pi-Alkyl	4.875	
			Arg491	Alkyl	4.094	
			His494	Pi-Pi T-Shaped	4.139	
				Pi-Alkyl	5.076	
			Ile497	Alkyl	5.259	
			Phe523	Pi-Alkyl	4.176	
			Leu560	Pi-Alkyl	5.115	
				Alkyl	4.254	
			Leu564	Alkyl	4.066	
					4.406	

			Leu575	Pi-Alkyl	5.391	
		Electrostatic interactions	Arg396	Pi-Cation	4.894	
			Glu399	Pi-Anion	3.536	
					3.585	
			Arg491	Pi-Cation	4.526	
TPO_{1-933} MT1	–3.2	Hydrogen Bond	His239	Conventional Hydrogen bond	2.209	12
			His494	Carbon Hydrogen bond	2.809	
		Hydrophobic interactions	His239	Pi-Alkyl	4.919	
			Phe243	Pi-Pi T-Shaped	4.875	
			Arg396	Alkyl	3.979	
				Pi-Alkyl	3.680	
					4.706	
			Phe524	Pi-Alkyl	3.864	
					4.435	
			Leu567	Alkyl	5.123	
				Pi-Alkyl	4.565	
					5.397	
TPO_{1-933} MT2	–11.5	Hydrogen Bond	His239	Conventional Hydrogen bond	3.155	20
			Arg491		2.743	
			Arg582		2.999	
					1.899	
		Hydrophobic interactions	His239	Pi-Pi T-Shaped	5.261	
			Val400	Alkyl	3.558	
			Arg491	Alkyl	4.362	
			His494	Pi-Alkyl	4.944	
					5.043	
			Ile497	Alkyl	5.116	
			Phe523	Pi-Alkyl	4.003	
					5.001	
			Leu564	Alkyl	3.729	
					5.453	
				Pi-Alkyl	5.391	
			Val566	Alkyl	4.717	
			Leu575	Pi-Alkyl	4.676	
		Electrostatic interactions	Arg396	Pi-Cation	4.176	
			Glu399	Pi-Anion	3.596	
					3.979	
TPO_{1-933} MT3	–7.9	Hydrogen Bond	Gln246	Conventional hydrogen bond	2.295	21
					2.351	
			Arg491		3.085	
			Ser568	Carbon hydrogen bond	1.969	
					3.043	
		Hydrophobic interactions	Phe243	Pi-Pi T-Shaped	5.806	
				Pi-Alkyl	5.488	
			Val400	Alkyl	5.463	

			Arg491	Alkyl	4.445	
			His494	Pi-Alkyl	4.209	
					4.567	
			Ile497	Alkyl	4.875	
			Phe523	Pi-Alkyl	4.544	
			Phe524	Pi-Alkyl	5.488	
			Leu560	Alkyl	4.702	
			Leu564	Alkyl	4.943	
					4.317	
			Val566	Alkyl	3.745	
			Leu575	Pi-Alkyl	4.767	
		Electrostatic interactions	Glu399	Pi-Anion	4.081	
					3.653	

The amino acid residues and their positions are designated as the three letter abbreviations and the corresponding number. WT = Wild type; MT1 = Mutant 1 (p.Ala373Ser); MT2 = Mutant 2 (p.Ser398Thr); MT3 = Mutant 3 (p.Thr725Pro).

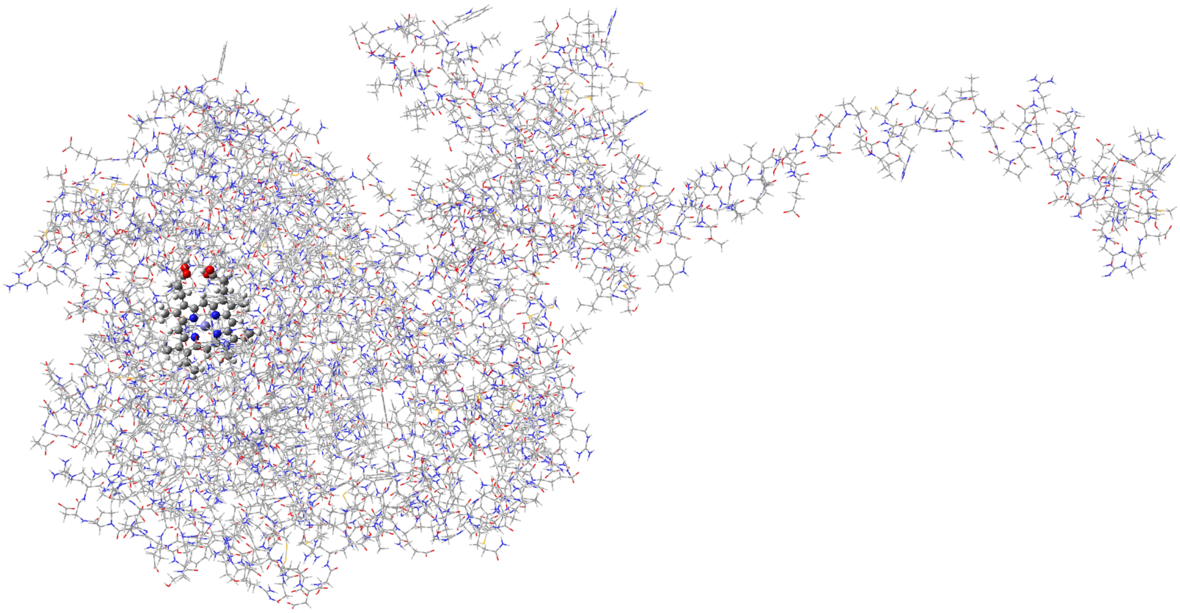

Supplementary Figure 3: QM/MM ONIOM calcualtion set up for protein-ligand complexes, high level (ball and stick) and low level (line).
